# van der Waals interaction energies for ferric ion sensors using prismatic and cylindrical pore approximations for a MOF pore

**DOI:** 10.1098/rsos.230232

**Published:** 2023-05-31

**Authors:** K. I. Louw, B. H. Bradshaw-Hajek, J. M. Hill

**Affiliations:** UniSA STEM, University of South Australia, Mawson Lakes, South Australia 5095, Australia

**Keywords:** Lennard–Jones interactions, van der Waals interactions, metal organic framework, continuum modelling, rectangular prism

## Abstract

Using the Lennard–Jones potential, we determine analytical expressions for van der Waals interaction energies between a point and a rectangular prism-shaped pore, writing them in terms of standard elementary functions. The parameter values for a new ferric ion sensor are used to compare these calculations with the cylindrical pore approximation for the interactions between an ion and a metal organic framework (MOF) pore. The results using the prismatic pore approximation predict the same qualitative outcomes as a cylindrical pore approximation. However, the prismatic approximation predicts lower magnitudes for both the interaction potential energy minimum and the force maximum, since the average distance from the centre-line to the surface of the prism is greater. We suggest that in some circumstances it is sufficient to use the simpler cylindrical approximation, provided that the cylinder radius is chosen so that the cross-sectional area is equal to the area of the prism pore opening. However, atoms at the nodes should remain approximated by semi-infinite lines. We also determine the interaction between a second ferric ion and a blocked MOF pore; as expected, the second ferric ion experiences a force away from the pore, implying that approaching ferric ions can only occupy vacant MOF pores.

## 1. Introduction

The van der Waals force is a weak non-bonded force between atoms and/or molecules. There are two basic approaches to calculate the interaction forces between two atoms or between two molecules [1,2]. We may adopt either a discrete method where forces are calculated for every non-bonded atom pair (commonly adopted by molecular dynamics), or use a continuum method to approximate the non-bonded atoms (or molecules) as geometric shapes, and then calculate the interaction energy between each interacting body [3,4]. In this paper, we use the second approach and the Lennard–Jones potential to calculate the van der Waals energies and forces between interacting molecules.

A good first step to approximating the interactions between molecules and porous materials is to consider them as points, spheres and cylinders (that is, simple geometric shapes with rotational symmetry). However, not all molecular structures possess rotational symmetry and while the use of geometric shapes with rotational symmetry results in simplified expressions for the interaction energies, the use of more representative shapes, for example, rectangular prisms, results in more complicated expressions. Baowan & Thamwattana [5] studied the absorption of water molecules into a silica gel by comparing three different approximate pore shapes: cylindrical, square prismatic and conical. Baowan & Hill [6] found expressions for the interaction energy of a point with a line, a plane, a ring, a spherical surface and a cylindrical surface. In these papers, the interactions are found in terms of series expansions and hypergeometric functions; expressions that are often considerably more complicated than those required in the case of symmetric approximations of molecular structures.

In this paper, we compare the calculation for a rectangular prism pore approximation to a cylindrical pore approximation. We base our formulation on that of Baowan & Thamwattana [5] to derive the interaction energy between a point and a rectangular prism, however, we evaluate analytically all the relevant integrals and express them in terms of standard functions. This work is motivated by its application to a new ferric ion sensor which makes use of the van der Waals interactions between ferric ions and an europium-based metal organic framework (MOF). The MOF possesses pores that in practice have an approximately parallelogram-shaped cross-section, however in previous models have been approximated by a cylinder [7]. In this paper, we determine a continuum approximation for the interaction of ferric ions with a MOF pore using the Lennard–Jones potential and a rectangular prism pore approximation, and explore the appropriateness of the simpler cylindrical pore approximation.

In §2, we describe the calculation of the van der Waals interaction energies and forces using the Lennard–Jones potential. Section 3 describes the physical structure of the example MOF and the geometrical approximations used in the continuum model. The main results for the prism pore approximation are presented and discussed in §4. Section 5 compares the appropriateness of the cylindrical pore approximation and presents further insight into sensor behaviour.

## 2. Continuum approximation for prismatic pore

**Figure 1.**
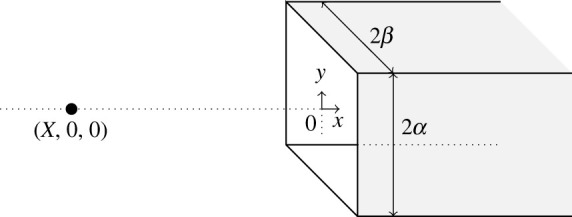
Schematic showing point particle and semi-infinite rectangular prism.

Figure 1 shows a point particle located on the centre-line of a semi-infinite rectangular prism. We assume the particle lies on the axis because the interaction energy has a global minimum when the interacting molecule does not deviate from the pore’s centre-line [8]. The centre-line of the prism is positioned along the *x*-axis and the entrance of the prism is located at the origin. The point particle initially has the coordinates (*X*, 0, 0), where *X* < 0 and the prism is formed by the four planes given by {(*x*_*p*_, *y*_*p*_, *z*_*p*_)|*x*_*p*_ ∈ [0, ∞), *y*_*p*_ = ±*α*, *z*_*p*_ ∈ [− *β*, *β*]} and {(*x*_*p*_, *y*_*p*_, *z*_*p*_)|*x*_*p*_ ∈ [0, ∞), *y*_*p*_ = [− *α*, *α*], *z*_*p*_ = ±*β*}, where *α* and *β* are real constants.

We use the Lennard–Jones potential to calculate the van der Waals interaction energies and forces. The Lennard–Jones potential, *U*(*ρ*), between two atoms is given by [9,10]

2.1 $$U(\rho )=-\frac{A}{\rho^{6}}+\frac{B}{\rho^{12}},$$

where *ρ* is the distance between the two interacting atoms, and *A* and *B* are the attractive and repulsive constants, respectively, for the two particular interacting atoms. We characterize the semi-infinite prism by four planes (walls) and four lines (edges). The interaction energy between a point and a surface *S* (the wall or an edge of the prism) with an assumed uniformly distributed atomic surface density *n* is given by

2.2 $$E(\rho )=n\int_{S}U(\rho )\;dS,$$

where *U*(*ρ*) is the Lennard–Jones potential (2.1).

The interaction energy between a point particle and one semi-infinite line (representing any atoms that might be located along an edge of the prism) is given by

2.3 $$E_{\mathrm{line}}(X)=n\int_{0}^{\infty }\left[-\frac{A}{{\left({\left(x-X\right)}^{2}+\alpha^{2}+\beta^{2}\right)}^{3}}+\frac{B}{{\left({\left(x-X\right)}^{2}+\alpha^{2}+\beta^{2}\right)}^{6}}\right]\;dx,$$

where the semi-infinite line is one of the edges of the prism described at the beginning of this section, and *ρ*^2^ = (*x* − *X*)^2^ + *α*^2^ + *β*^2^. Details for the evaluation of this integral are provided in appendix A.

The interaction energy between a point particle and one vertical semi-infinite plane (that is, where *z*_*p*_ = ±*β*) is given by

2.4 $$\begin{array}{rl} E_{\mathrm{vert}}(X) & =n\int_{0}^{\infty }\int_{-\alpha }^{\alpha }\left[-\frac{A}{{\left({\left(x-X\right)}^{2}+y^{2}+\beta^{2}\right)}^{3}}+\frac{B}{{\left({\left(x-X\right)}^{2}+y^{2}+\beta^{2}\right)}^{6}}\right]\;dy\;dx,\\ & =2n\int_{0}^{\infty }\int_{0}^{\alpha }\left[-\frac{A}{{\left({\left(x-X\right)}^{2}+y^{2}+\beta^{2}\right)}^{3}}+\frac{B}{{\left({\left(x-X\right)}^{2}+y^{2}+\beta^{2}\right)}^{6}}\right]\;dy\;dx, \end{array}$$

where the coordinates of the vertical semi-infinite plane are given by (*x*, *y*, ± *β*), *x* ∈ [0, ∞), *y* ∈ [−*α*, *α*] and *ρ*^2^ = (*x* − *X*)^2^ + *y*^2^ + *β*^2^. Details for the evaluation of this integral are provided in appendix B. Similarly, the interaction energy between a point particle and one horizontal semi-infinite plane is given by

$$E_{\mathrm{hori}}(X)=2n\int_{0}^{\infty }\int_{0}^{\beta }\left[-\frac{A}{{\left({\left(x-X\right)}^{2}+\alpha^{2}+z^{2}\right)}^{3}}+\frac{B}{{\left({\left(x-X\right)}^{2}+\alpha^{2}+z^{2}\right)}^{6}}\right]\;dz\;dx,$$

where the coordinates of the horizontal semi-infinite plane are given by (*x*, ± *α*, *z*), *x* ∈ [0, ∞), *z* ∈ [−*β*, *β*] and *ρ*^2^ = (*x* − *X*)^2^ + *α*^2^ + *z*^2^.

The total interaction energy is given by the sum of the interaction energies between the point and the components of the prism (i.e. the four lines and four planes). As part of this calculation, we determine the interaction energy between the point and the various atom types located on each surface.

The van der Waals force in the axial direction is the component of the derivative of the interaction energy that is directed along the axis of symmetry. It can be calculated using similar triangles [11]

$$F(X)=\frac{x-X}{\rho }\frac{dE}{d\rho },$$

where *E* is the interaction energy, defined in equation (2.2). The interaction force between a point particle and one semi-infinite line is given by

$$F_{\mathrm{line}}(X)=n\left[\frac{A}{{\left(X^{2}+\alpha^{2}+\beta^{2}\right)}^{3}}-\frac{B}{{\left(X^{2}+\alpha^{2}+\beta^{2}\right)}^{6}}\right].$$

The interaction force between a point particle and one vertical semi-infinite plane is given by

$$\begin{array}{rl} F_{\mathrm{vert}}(X) & =2n\{\frac{A}{8}[\frac{2\alpha }{{\left(\alpha^{2}+\beta^{2}+X^{2}\right)}^{2}(\beta^{2}+X^{2})}+\frac{3\alpha }{(\alpha^{2}+\beta^{2}+X^{2}){\left(\beta^{2}+X^{2}\right)}^{2}}\\ & \;+\;3{\left(\beta^{2}+X^{2}\right)}^{-5/2}\arctan\left(\frac{\alpha }{{\left(\beta^{2}+X^{2}\right)}^{1/2}}\right)]\\ & \;-\frac{B}{1280}[\frac{128\alpha }{{\left(\alpha^{2}+\beta^{2}+X^{2}\right)}^{5}(\beta^{2}+X^{2})}+\frac{144\alpha }{{\left(\alpha^{2}+\beta^{2}+X^{2}\right)}^{4}\;{\left(\beta^{2}+X^{2}\right)}^{2}}\\ & \;+\frac{168\alpha }{{\left(\alpha^{2}+\beta^{2}+X^{2}\right)}^{3}{\left(\beta^{2}+X^{2}\right)}^{3}}+\frac{210\alpha }{{\left(\alpha^{2}+\beta^{2}+X^{2}\right)}^{2}\;{\left(\beta^{2}+X^{2}\right)}^{4}}\\ & \;+\frac{315\alpha }{(\alpha^{2}+\beta^{2}+X^{2}){\left(\beta^{2}+X^{2}\right)}^{5}}+\;315{\left(\beta^{2}+X^{2}\right)}^{-11/2}\arctan\left(\frac{\alpha }{{\left(\beta^{2}+X^{2}\right)}^{1/2}}\right)]\}. \end{array}$$

The interaction force between a point particle and one horizontal semi-infinite plane is given by

$$\begin{array}{rl} F_{\mathrm{hori}}(X) & =2n\{\frac{A}{8}[\frac{2\beta }{{\left(\alpha^{2}+\beta^{2}+X^{2}\right)}^{2}(\alpha^{2}+X^{2})}+\frac{3\beta }{(\alpha^{2}+\beta^{2}+X^{2}){\left(\alpha^{2}+X^{2}\right)}^{2}}\\ & \;+\;3{\left(\alpha^{2}+X^{2}\right)}^{-5/2}\arctan\left(\frac{\beta }{{\left(\alpha^{2}+X^{2}\right)}^{1/2}}\right)]\\ & \;-\frac{B}{1280}[\frac{128\beta }{{\left(\alpha^{2}+\beta^{2}+X^{2}\right)}^{5}(\alpha^{2}+X^{2})}+\frac{144\beta }{{\left(\alpha^{2}+\beta^{2}+X^{2}\right)}^{4}{\left(\alpha^{2}+X^{2}\right)}^{2}}\\ & \;+\frac{168\beta }{{\left(\alpha^{2}+\beta^{2}+X^{2}\right)}^{3}{\left(\alpha^{2}+X^{2}\right)}^{3}}+\frac{210\beta }{{\left(\alpha^{2}+\beta^{2}+X^{2}\right)}^{2}{\left(\alpha^{2}+X^{2}\right)}^{4}}\\ & \;+\frac{315\beta }{(\alpha^{2}+\beta^{2}+X^{2}){\left(\alpha^{2}+X^{2}\right)}^{5}}+315{\left(\alpha^{2}+X^{2}\right)}^{-11/2}\arctan\left(\frac{\beta }{{\left(\alpha^{2}+X^{2}\right)}^{1/2}}\right)]\}. \end{array}$$

The total interaction force is given by the sum of the interaction forces between the point and the components of the prism.

In this section, we have presented expressions for the interaction energy and force between a point particle and the components of a rectangular pore. These investigations are motivated by a newly developed ferric ion sensor which we describe in the following section.

## 3. Application to ferric ion sensor

Up-to-date knowledge of the ferric ion concentration in minerals leaching processes is essential to maximize copper and uranium recovery. Current methods of ferric ion sensing are expensive and both time and energy consuming which leads to waste and operation inefficiencies. Xu *et al.* [12] reported the sensing properties of an europium-based MOF, and Rozenberga *et al.* [13] reassessed the reported europium-based MOF structure and discovered that the methoxy group remains after the MOF synthesization process. The parameters and results given in this section correspond to the compound named EuBDC-OMe which is a crystalline MOF structure [13].

The ferric ion sensor used in the minerals leaching process is exposed to solutions with very low pH levels, and this highly acidic environment results in almost insignificant double layers. Accordingly, the Coulomb potential becomes inconsequential and only the van der Waals interactions are studied.

**Figure 2.**
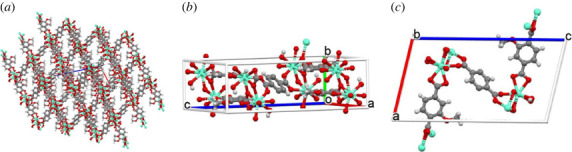
(*a*) Atomic structure of EuBDC-OMe, with metallic europium nodes as turquoise spheres, and organic linkers with oxygen as red spheres, hydrogen white and carbon grey [13]. (*b*) EuBDC-OMe unit cell, building block for crystalline structure. (*c*) EuBDC-OMe unit cell along *b*-axis of symmetry.

Figure 2*a* shows the three-dimensional view of the MOF crystal structure and pores. In order to investigate the van der Waals interaction between a point ferric ion and the MOF crystal, we consider a single isolated pore. The MOF pore is a parallelogram prism which we approximate by a rectangular prism, where the ligands are modelled as semi-infinite planes and the europium nodes with associated water molecules as semi-infinite lines. The semi-infinite pore approximation for the interactions calculated below gives a good approximation for a finite pore of depth 70 Å or more (Rozenberga *et al*. [13] report that the MOF crystal sizes are of the order of 100 nm). In figure 2*b*, the opposite faces of the unit cell are comprised of two ligands (where the benzene rings are facing each other) bridging four europium metal ions (along the *a*-axis of symmetry), and the adjacent sides have a single diagonal ligand.

Each unit cell has a molecular formula of Eu_2_(C_9_H_6_O_5_)_3_ · 2H_2_O and these unit cells are repeated to create the MOF structure shown in figure 2*a*. We assume that a single pore consists of two of these molecular unit cells together with four linking europium atoms and water molecules [13]. The europium nodes with associated water molecules are located along the edges of the prism, and so we approximate the europium ions and associated water molecules by four semi-infinite lines. The bridging ligands are approximated by four semi-infinite planes. The vertical planes are parallel to the *a*-axis of symmetry. The numbers of each atom type and other MOF parameters are given in table 1.

**Table 1.** Physical parameters for single EuBDC-OMe pore used in §4 [13].

atomic type	number per single pore/cell				
	atoms at node	nodes	atoms in ligand	ligands	total atoms
hydrogen	4	4	6	6	52
carbon	0	0	9	6	54
oxygen	2	4	5	6	38
europium	2	4	0	0	8

MOF parameters	
*a*-axis basis vector	11.513 Å
*b*-axis basis vector	6.829 Å
*c*-axis basis vector	20.976 Å
*a*-axis and *c*-axis intersection angle	*θ* = 102.8°

The width of the prism is taken as half the magnitude of the *c*-axis basis vector (in table 1), so that 2*β* = 20.976/2 = 10.488 Å, and the adjacent side of the parallelogram has the magnitude of the *a*-axis basis vector. Therefore, the length of the prism is 2*α* = 11.513 × sin (180 − *θ*) = 11.2269 Å. This approximation ensures that the area of the rectangle is equal to the area of the parallelogram. Note that the vertical plane has two bridging ligands (parallel to the *a*-axis basis vector) and that the mean surface density for the vertical plane should be about twice the value of the horizontal plane, see table 2.

**Table 2.** Mean density for each atom type for approximated prismatic MOF unit cell.

atomic type	mean surface density, *n*_*k*_		
	line (Å^−1^)	vertical plane (Å^−2^)	horizontal plane (Å^−2^)
europium	0.2929	0	0
hydrogen	0.5857	0.1565	0.0838
oxygen	0.2929	0.1304	0.0698
carbon	0	0.2348	0.1257

To calculate the mean surface density, we divide the number of atoms by the surface area of each component of a single approximated pore (lines/planes). For example, the mean surface density of hydrogen atoms at the nodes (lines) is the number of hydrogen atoms at a node divided by the length of the single approximated pore, i.e. 4/*b*-axis basis vector length. The mean surface density for hydrogen atoms found in the bridging ligands parallel to the *c*-axis basis vector (the horizontal plane), is 6/(2*β* × *b*-axis basis vector length). This is calculated in a similar way for the hydrogen atoms on the adjacent side of the prism (the vertical plane).

Ferric ions are typically associated with six water molecules when in solution [14]. However, we only consider the interaction between the ferric ion (without associated water molecules) and the MOF pore. In terms of the van der Waals interactions, inclusion of the water molecules increases the magnitude of the interaction energy and force, but it does not change its qualitative behaviour [7]. Accordingly, for comparison with the cylindrical pore approximation we ignore the effect of the water molecules, and compare the interactions of the ferric ion only with the two pore shapes. We note that the presence of associated water molecules increases the likelihood of any steric interactions. The ferric ion is modelled as a point.

To calculate the total interaction energy between the ferric ion point and the semi-infinite prism MOF pore, we sum all non-bonded interactions between the ferric ion point and each atom type found in the MOF pore. That is,

$$E_{\mathrm{Fe},\mathrm{pore}}=E_{\mathrm{Fe},H}+E_{\mathrm{Fe},O}+E_{\mathrm{Fe},C}+E_{\mathrm{Fe},\mathrm{Eu}}=\sum_{k}E_{\mathrm{Fe},k}.$$

In this example, the MOF pore is characterized as four planes and four lines to represent the bridging ligands and nodes of the MOF pore. The interaction energies between the ferric ion and each atom type in the MOF pore are calculated as follows. Europium atoms in the MOF pore are only located at the nodes of the MOF pore, which are approximated by four semi-infinite lines. The interaction energy between the ferric ion point and the europium nodes is given by

$$E_{\mathrm{Fe},\;\mathrm{Eu}}=4E_{\mathrm{line}}(\rho ),$$

where *ρ*^2^ = (*x* − *X*)^2^ + *α*^2^ + *β*^2^. Carbon atoms in the MOF pore are only located in the bridging ligands of the MOF pore, which are approximated by four semi-infinite planes (two vertical and two horizontal). The interaction energy between the ferric ion point and the carbon atoms in the MOF ligands is given by

$$E_{\mathrm{Fe},C}=2E_{\mathrm{vert}}(\rho_{1})+2E_{\mathrm{hori}}(\rho_{2}),$$

where $\rho_{1}^{2}={\left(x-X\right)}^{2}+y^{2}+\beta^{2}$ and $\rho_{2}^{2}={\left(x-X\right)}^{2}+\alpha^{2}+z^{2}$. Note that the mean surface densities, *n*, for the vertical and horizontal planes differ and need to be considered accordingly. Hydrogen atoms are located at the four nodes of the MOF pore (water molecules associated with the europium atoms) and in the bridging ligands. The interaction energy between the ferric ion point and the hydrogen atoms is given by

$$E_{\mathrm{Fe},H}=4E_{\mathrm{line}}(\rho )+2E_{\mathrm{vert}}(\rho_{1})+2E_{\mathrm{hori}}(\rho_{2}).$$

Similarly, the interaction energy between the ferric ion point and the oxygen atoms in the MOF ligands and at the MOF nodes is given by

$$E_{\mathrm{Fe},O}=4E_{\mathrm{line}}(\rho )+2E_{\mathrm{vert}}(\rho_{1})+2E_{\mathrm{hori}}(\rho_{2}).$$

The total interaction force between the ferric ion and the semi-infinite prism MOF pore is given by

$$F_{\mathrm{Fe},\mathrm{pore}}=F_{\mathrm{Fe},H}+F_{\mathrm{Fe},O}+F_{\mathrm{Fe},C}+F_{\mathrm{Fe},\mathrm{Eu}}=\sum_{k}F_{\mathrm{Fe},k},$$

where

$$F_{\mathrm{Fe},\mathrm{pore}}=\frac{x-X}{\rho }\frac{dE_{\mathrm{Fe},\mathrm{pore}}}{d\rho }.$$

In the following section, we discuss our numerical findings.

## 4. Results and discussions

The Lennard–Jones parameters are given in table 3, where the attractive and repulsive constants can be calculated using $A=\epsilon \sigma^{6}$ and $B=\epsilon \sigma^{12}$. Note that the parameters provided in table 3 are for interactions between like atoms. The attractive and repulsive constants for the interaction between different atomic types can be calculated by re-evaluating the well depth and van der Waals diameter using the geometric and arithmetic means, respectively, that is $\epsilon_{ij}=\sqrt{\epsilon_{i}\epsilon_{j}}$ and *σ*_*ij*_ = (*σ*_*i*_ + *σ*_*j*_)/2 [6].

**Table 3.** Attractive and repulsive constants for Lennard–Jones parameters for interactions between like atoms [15].

atom type	van der Waals diameter, *σ* (Å)	well-depth, ϵ (eV)
hydrogen, H	2.5711	0.0019
carbon, C	3.4309	0.0045
oxygen, O	3.1181	0.0026
ferric ion, Fe^3+^	2.5943	0.0006
europium ion, Eu^3+^	3.1119	0.0003

### 4.1. van der Waals interaction energy and force

**Figure 3.**
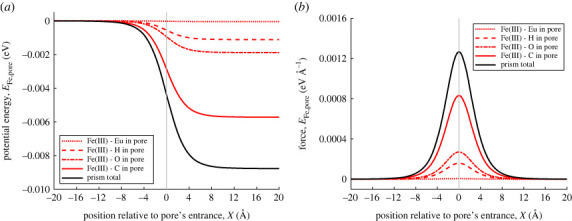
(*a*) van der Waals interaction energy between ferric ion point and MOF pore. Red dotted, dashed, dashed dotted and solid lines show interaction energies between ferric ion and different atom types in MOF pore. Vertical line marks location of pore opening. (*b*) van der Waals interaction force between ferric ion and MOF pore.

Figure 3 shows the individual van der Waals interaction energies and forces between a ferric ion and all atom types found in the MOF pore approximated by a rectangular prism. The europium nodes contribute the least to the interaction energy and force. This is attributed to europium having the fewest number of atoms in the pore and that europium atoms are only located at the edges of the prism. Since europium atoms are the furthermost atom types from the MOF pore centre-line, it stands that their contribution would be the smallest. In addition, europium has the smallest well-depth ϵ.

By contrast, the interaction energy and force between the ferric ion and carbon atoms in the MOF pore contributes the most to the total energy and force. There are 54 carbon atoms in a single representative MOF pore and the average carbon-ferric ion distance *ρ* is shorter than the average europium–ferric ion distance, therefore carbon has a greater influence when interacting with the ferric ion. In addition, carbon has the largest well-depth.

The total interaction energy indicates that it is favourable for the ferric ion to reside in the MOF pore, as the ferric ion experiences a potential energy minimum inside the MOF pore (figure 3*a*). The total interaction force is positive, indicating that there is force to the right acting on the ferric ion, pulling it towards the MOF pore entrance (figure 3*b*).

## 5. Prismatic versus cylindrical approximation

**Figure 4.**
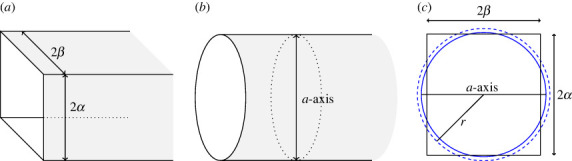
Approximation of EuBDC-OMe MOF pore (*a*) as semi-infinite rectangular prism and (*b*) as a semi-infinite cylinder. (*c*) Area difference between prism, original cylinder and fitted cylinder, depicted as solid black line, solid blue line and dashed blue line, respectively.

In this section, we compare results for two different approximations for the MOF pore: a rectangular prism and a cylinder. The expressions describing the interactions in the case of the cylinder (not shown here) are much simpler than the case of a rectangular prism due to its rotational symmetry. Here, we make comparisons with cylinders of two different radii. The first mirrors the results shown in a previous paper [7] where the diameter of the cylinder was taken to be the same length as the *a*-axis-basis vector (denoted hereon as the original cylindrical approximation). In the second, the radius of the cylinder is chosen so that the area of the cylindrical pore opening is the same as the opening for the rectangular prism, $r=2\sqrt{\alpha \beta /\pi }$ (denoted hereon as the fitted cylindrical approximation). Figure 4*a*,*b* shows the semi-infinite rectangular prism and semi-infinite cylindrical MOF pore approximations. Figure 4*c* shows the cross-section of each of the pores. Note that the fitted cylinder is larger than original cylinder.

We maintain the continuum approach, and therefore for the cylindrical MOF pore calculations, we assume that all atoms are ‘smoothed’ across the surface of the cylinder. Table 4 provides the mean densities that are required to calculate the interaction energy and force. Note that the calculations for a point interacting with a cylinder is not provided in this paper, and we refer the reader to Louw *et al*. [7].

**Table 4.** Parameter values used incorporating physical attributes for cylindrical and fitted cylindrical MOF pore.

geometric parameters		
approx. cylinder radius	*r* = 6.1221 Å	
atomic type	mean surface density, *n*_*k*_ (Å^−2^)	
	cylinder, radius 1/2 *a*-axis	approx. cylinder, radius *r*
europium	0.0324	0.0305
carbon	0.2186	0.2056
hydrogen	0.2105	0.1980
oxygen	0.1538	0.1447

**Figure 5.**
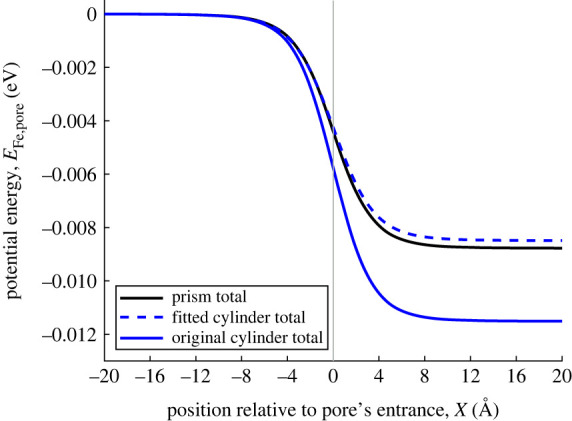
van der Waals interaction energy between ferric ion and MOF pore. Black line shows interaction energy between ferric ion and prism. Blue dashed and solid line show interaction energy between ferric ion and fitted cylindrical pore, ferric ion and original cylindrical pore, respectively. Vertical line marks location of pore opening.

Figure 5 compares the interaction energy between a ferric ion and the MOF pore, showing the rectangular approximation, together with both the original and fitted cylinder approximations. The magnitude of the predicted potential energy minimum is much greater for the original cylindrical approximation than for both the rectangular prism and the fitted cylindrical approximations. The fitted cylinder and the rectangular prism, having the same cross-sectional area, provide very similar predictions for the interaction energy, demonstrating that in this scenario, it is sufficient to use the simpler cylindrical formulation, providing that the cross-sectional areas match. In the following figures, we investigate in more detail the contributions of the planes and lines, and the different atom types.

**Figure 6.**
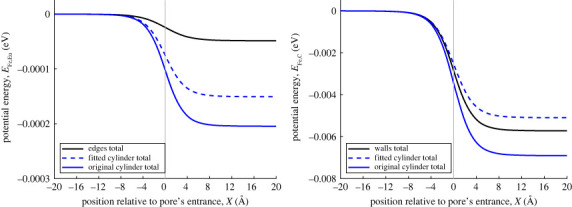
(*a*) van der Waals interaction energy between ferric ion and europium in pore. Black line shows interaction energy between ferric ion and prism. Blue dashed and solid line show interaction energy between ferric ion and fitted cylindrical pore, ferric ion and original cylindrical pore, respectively. Vertical line marks location of pore opening. (*b*) van der Waals interaction energy between ferric ion and carbon in pore.

The cylindrical approximations ignore the specific locations of the atoms and their respective distances from the *x*-axis. This is more problematic for the atoms located only along the edges of the rectangular prism (the lines), such as the europium atoms and their associated water molecules in this example. The cylindrical approximation assumes that all atoms are smoothed over the surface of the cylinder, which is closer to the *x*-axis than the edges of the rectangular prism. As a consequence, their contribution to the total interaction energy is much greater than it is in practice. Figure 6*a* shows the contribution of the ferric ion–europium interaction to the total potential energy. As expected, the europium contribution in the cylindrical approximations is much greater in magnitude than the rectangular prism approximation.

By contrast, the situation for atoms located along the sides of the rectangular prism is more nuanced. As shown in figure 4, sometimes the cylindrical approximation overestimates the distance between these atoms and the *x*-axis, while sometimes it underestimates the distance. In the case of the rectangular prism, the average distance of an atom on the vertical walls to the *x*-axis is given by

$$\begin{array}{rl} \frac{1}{2\alpha }\int_{-\alpha }^{\alpha }\sqrt{\beta^{2}+y^{2}}\;dy & =\frac{1}{2}\sqrt{\alpha^{2}+\beta^{2}}+\frac{\beta^{2}}{4\alpha }\ln\left[\frac{1}{\beta^{2}}\left(2\alpha^{2}+\beta^{2}+2\alpha \sqrt{\alpha^{2}+\beta^{2}}\right)\right]=6.1197\;Å. \end{array}$$

A corresponding expression can be derived for the average distance of an atom on the horizontal walls to the *x*-axis

$$\begin{array}{rl} \frac{1}{2\beta }\int_{-\beta }^{\beta }\sqrt{z^{2}+\alpha^{2}}\;dz & =\frac{1}{2}\sqrt{\alpha^{2}+\beta^{2}}+\frac{\alpha^{2}}{4\beta }\ln\left[\frac{1}{\alpha^{2}}\left(\alpha^{2}+2\beta^{2}+2\beta \sqrt{\alpha^{2}+\beta^{2}}\right)\right]=6.3468\;Å. \end{array}$$

Both these average distances are greater than the original cylinder’s radius, confirming that the original cylinder approximation would overestimate the magnitude of the interaction energy. The fitted cylinder’s radius is very close to the average distance of both the vertical and horizontal walls (less than 4% difference).

Even though there is a small difference between the fitted cylinder’s radius and the average distance between the centre-line and the vertical wall, there are twice as many atoms located on the vertical wall, so that the magnitude of the interaction energy for the walls is still underestimated when using the fitted cylinder approximation. The difference between the fitted cylinder radius and the average distance to the horizontal wall is not significant enough for the fitted cylinder approximation to overestimate the magnitude of the interaction energy. This further emphasizes the distance sensitivity of the interaction energy calculation, and the importance of accounting for atom surface density correctly. Although not shown here, there is a greater agreeability between the fitted approximation and a perfect square.

**Figure 7.**
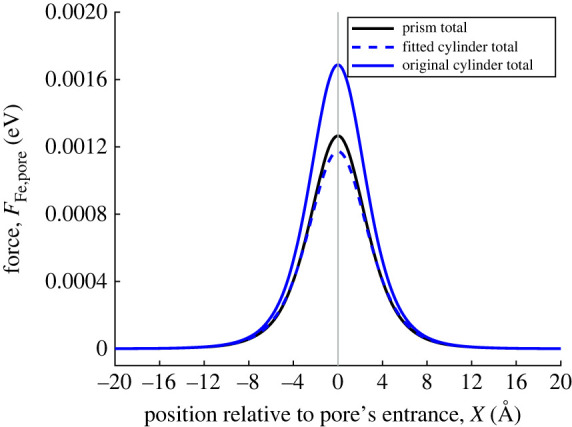
van der Waals interaction force between ferric ion and MOF pore. Black line shows interaction force between ferric ion and prism. Blue dashed and solid line show interaction force between ferric ion and fitted cylindrical pore, ferric ion and original cylindrical pore, respectively. Vertical line marks location of pore opening.

Figure 7 shows the interaction force between a ferric ion and the three different approximations of the MOF pore, and reflects the findings discussed above for the interaction energy. The original cylindrical approximation overestimates the magnitude of the force in comparison to the rectangular prism approximation, however the fitted cylinder and rectangular prism approximations (with the same cross-sectional area) are much closer in magnitude.

**Figure 8.**
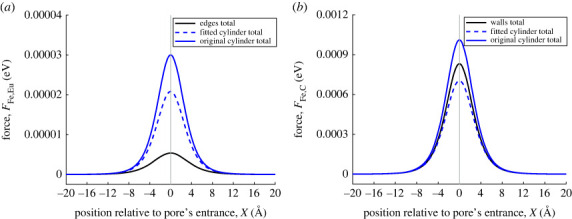
(*a*) van der Waals interaction force between ferric ion and europium in pore. Black line shows interaction force between ferric ion and prism. Blue dashed and solid lines show interaction force between ferric ion and fitted cylindrical pore, ferric ion and original cylindrical pore, respectively. Vertical line marks location of pore opening. (*b*) van der Waals interaction force between ferric ion and carbon in pore.

In the same way as before, we compare the contributions of the europium and carbon atoms for the three different approximations. Figure 8 echoes the findings from figure 6. Both cylindrical approximations overpredict the contribution of the europium ions because the radius of both cylinders in less than the distance from the prism edge to the *x*-axis. The same argument is true for the original cylinder approximation overpredicting the contribution of carbon atoms. The fitted cylinder underpredicts the contribution of carbon atoms mainly due to its radius being less than the average horizontal distance.

One important finding to note is that regardless of the shape used to approximate the MOF pore (rectangular prism or cylindrical), the force on the ferric ion is always directed to the right, indicating that the ferric ion will be attracted to the MOF pore entrance.

### 5.1. Implications of a pore blockage for sensing abilities

In a previous paper, we proposed that a hydrated ferric ion is geometrically too large to fit inside the europium MOF pores due to steric interactions and as a consequence it will be attracted to the MOF pore, but will remain at the entrance without entering [7]. Subsequently, these results were confirmed experimentally by Rozenberga *et al.* [13]. Therefore, in this section we consider the interaction between a ferric ion and a MOF pore where the entrance is already occupied by another ferric ion. We use the rectangular prism approximation.

**Figure 9.**
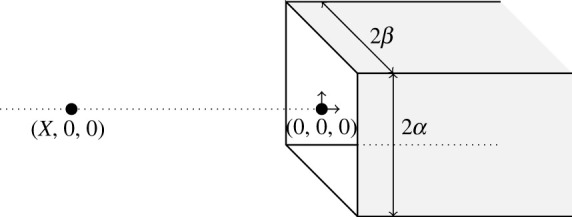
Schematic showing point particle and blocked semi-infinite rectangular prism by point particle.

This subsection maintains the geometry where the MOF pore is approximated by a semi-infinite prism, and is described in figure 1 and the surrounding text. In addition, we assume that the MOF pore entrance is blocked by a ferric ion (not hydrated), where the Cartesian coordinates of the blocked ferric ion are (0, 0, 0), as shown in Figure 9.

**Figure 10.**
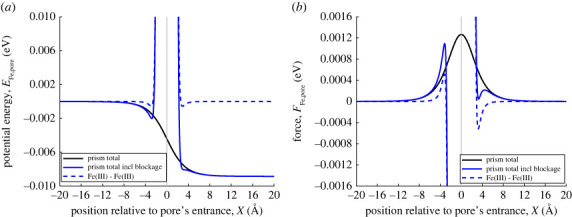
(*a*) van der Waals interaction energy between (unhydrated) ferric ion and blocked MOF pore. Blue dashed and solid line show interaction energy between two ferric ions, and ferric ion and blocked MOF pore, respectively. Vertical line marks location of pore opening. (*b*) van der Waals interaction force between ferric ion and blocked MOF pore.

Figure 10*a* shows that there is a local potential energy minimum at 2.85 Å to the left of the pore entrance. A ferric ion farther away from the pore entrance experiences a force to the right (towards the MOF pore), while a ferric ion closer to the entrance will experience a strong repulsive force (figure 10*b*). The potential energy minimum just outside the pore entrance is much smaller in magnitude than that of a vacant pore (compare with the minimum of the black line in figure 10*a*), while the magnitude of the repulsive force close to the blocked pore is very large. As a consequence, in practice it is unlikely that a ferric ion will remain at the local potential energy minimum outside the pore, rather it will more likely move to occupy a vacant MOF pore, which has the global minimum energy. This means that more ferric ions will become associated with the MOF crystal, which in turn is likely to improve sensor performance.

## 6. Conclusion

In this paper, we have compared the Lennard–Jones interaction energy between a point and a pore, as calculated using the simpler and more typical cylindrical approximation and the more complicated rectangular prism approximation. Building on a previous proposal, we have evaluated all the relevant integrals analytically. By contrast, analytical expressions had previously only been derived for the cylindrical approximation.

The motivation of the present work is the determination of an optimal pore approximation for a new ferric ion sensor that uses europium-based MOF crystals. Using parameter values relevant for this particular example, we determine the van der Waals interactions between a ferric ion and the MOF pore and use the results to compare the cylindrical and the rectangular prism approximations of the pore.

Numerical values for the prismatic MOF pore approximation indicate that the minimum potential energy of the ferric ion occurs inside the MOF pore; namely that the inside of the MOF pore is the most energetically favourable position for the ferric ion to reside. Further, numerical values for the interaction force indicate that the ferric ion experiences a force of greatest magnitude at the MOF pore entrance. The force is directed towards the MOF pore.

While the analytical expressions for the interaction energy between a point and a prism are much more complex than those between a point and a cylinder, the interaction energies and forces have the same qualitative behaviour regardless of the approximation (cylindrical or rectangular prism). The qualitative match is closest when the approximations have the same cross-sectional area.

Any differences between the cylindrical and the rectangular prism approximations can be understood by considering the distance between particular atoms in the pore structure and the centre-line of the pore. For example, for the MOF pore considered here, the atoms located along the edges of the prism make a smaller contribution than the same atoms in the cylindrical approximation (where they are assumed to lie on the surface of the cylinder and as such are closer to the centre-line). As a consequence, in the general case, it is important to consider whether any such atoms should be considered separately, particularly if their Lennard–Jones pair potential parameters (*σ* and ϵ) are large compared with other atom types in the system.

We have also considered the interactions between a ferric ion and a blocked MOF pore. Our results indicate that the second ferric ion experiences strong interaction forces directed away from the MOF pore at distances closer than 2.8 Å from the MOF pore entrance. As a consequence, it might be expected that the second ferric ion moves towards an alternative unoccupied pore, increasing the proportion of associated ferric ions and the sensitivity of the ferric ion sensor.

This paper uses a rectangular prism to provide an improved approximation to a parallelogram MOF pore. A rectangular prism was chosen since it maintains some of the features of a parallelogram (four sides and edges, same base length and cross-sectional area), while still being mathematically tractable. Further analysis would be required to derive analytical expressions for the interactions between a point and a slanted plane to compare how well the rectangular prism approximates a parallelogram prism.

## Data Availability

This article has no additional data.

## Appendix A. Interaction between a point and a line (evaluation of integral (2.3))

In this appendix, we determine an analytical expression for the interaction energy between a point and a semi-infinite line. When considering the interaction energies in a three-dimensional Cartesian coordinate frame, between a point (*X*, 0, 0) and a semi-infinite line (*x*_*p*_, *y*_*p*_, *z*_*p*_), the Lennard–Jones potential is integrated along the semi-infinite line

$$E=n\int_{0}^{\infty }\left[-\frac{A}{{\left({\left(x-X\right)}^{2}+\alpha^{2}+\beta^{2}\right)}^{3}}+\frac{B}{{\left({\left(x-X\right)}^{2}+\alpha^{2}+\beta^{2}\right)}^{6}}\right]\;dx,$$

where (*x*_*p*_, *y*_*p*_, *z*_*p*_) = (*x*, *α*, *β*), where *x* ∈ [0, ∞), *α* and *β* are real constants, and *ρ*^2^ = (*x* − *X*)^2^ + *α*^2^ + *β*^2^. Let $x_{*}=x-X$, then at the lower bound of integration becomes *x* = 0, $x_{*}=-X$, and at the upper bound of integration, *x* → ∞ becomes $x_{*}\to \infty$, so that

$$E=n\int_{-X}^{\infty }\left[-\frac{A}{{\left(x_{*}^{2}+\alpha^{2}+\beta^{2}\right)}^{3}}+\frac{B}{{\left(x_{*}^{2}+\alpha^{2}+\beta^{2}\right)}^{6}}\right]\;dx_{*}.$$

Let *λ*^2^ = *α*^2^ + *β*^2^ and $x_{*}=\lambda \tan\psi$, then $\lambda^{2}+x_{*}^{2}=\lambda^{2}+\lambda^{2}\tan^{2}\psi =\lambda^{2}\sec^{2}\psi$ and $dx_{*}=\lambda \sec^{2}\psi \;d\psi$. The integration limits are transformed as follows: as $x_{*}\to \infty$, *ψ* = *π*/2 and when $x_{*}=-X$, $\psi =\psi_{0}=\arctan[-X/\lambda ]$. We then obtain

$$E=n\lambda^{-5}\int_{\psi_{0}}^{\pi /2}[-A\cos^{4}\psi +\lambda^{-6}B\cos^{10}\psi ]\;d\psi .$$

The potential energy of a point interacting with a semi-infinite line can therefore be written as

A 1 $$E=n\lambda^{-5}[-AJ_{2}(\lambda ,-X)+\lambda^{-6}BJ_{5}(\lambda ,-X)],$$

where *J*_*n*_(*λ*, −*X*) is the definite integral of powers of cosine functions and the general form of *J*_*n*_(*λ*, −*X*) is given by

$$J_{n}(\lambda ,-X)=\int_{\theta }^{\pi /2}\cos^{2n}\psi \;d\psi .$$

For expression (A 1), we require *J*_2_(*λ*, − *X*) and *J*_5_(*λ*, −*X*) which can be evaluated respectively as,

$$\begin{array}{rl} J_{2}(\lambda ,-X) & =\int_{\theta }^{\pi /2}\cos^{4}\psi \;d\psi ,\\ & =\frac{3}{8}\cdot \frac{\pi }{2}+\left[\frac{1}{4}\frac{X\lambda^{3}}{{\left(\lambda^{2}+X^{2}\right)}^{2}}+\frac{3}{8}\frac{X\lambda }{\left(\lambda^{2}+X^{2}\right)}+\frac{3}{8}\arctan\left(\frac{X}{\lambda }\right)\right]. \end{array}$$

and

$$\begin{array}{rl} J_{5}(\lambda ,-X) & =\int_{\theta }^{\pi /2}\cos^{10}\psi \;d\psi ,\\ & =\frac{63}{256}\cdot \frac{\pi }{2}+[\frac{1}{10}\frac{X\lambda^{9}}{{\left(\lambda^{2}+X^{2}\right)}^{5}}+\frac{9}{80}\frac{X\lambda^{7}}{{\left(\lambda^{2}+X^{2}\right)}^{4}}+\frac{21}{160}\frac{X\lambda^{5}}{{\left(\lambda^{2}+X^{2}\right)}^{3}}\\ & \;+\frac{21}{128}\frac{X\lambda^{3}}{{\left(\lambda^{2}+X^{2}\right)}^{2}}+\frac{63}{256}\frac{X\lambda }{\left(\lambda^{2}+X^{2}\right)}+\frac{63}{256}\arctan\left(\frac{X}{\lambda }\right)], \end{array}$$

where tan*θ* = −*X*/*λ*.

## Appendix B. Interaction between a point and a plane (evaluation of integral (2.4))

In this appendix, we evaluate the interaction energy between a point and a semi-infinite plane in order to express it in terms of elementary functions. When considering the interacting energies in a three-dimensional Cartesian coordinate frame, between a point (*X*, 0, 0) and a semi-infinite plane (*x*_*p*_, *y*_*p*_, *z*_*p*_), the Lennard–Jones potential is integrated only along the surface of the semi-infinite plane,

$$E=n\int_{0}^{\infty }\int_{\alpha_{1}}^{\alpha_{2}}\left[-\frac{A}{{\left({\left(x-X\right)}^{2}+y^{2}+\beta^{2}\right)}^{3}}+\frac{B}{{\left({\left(x-X\right)}^{2}+y^{2}+\beta^{2}\right)}^{6}}\right]\;dy\;dx,$$

where (*x*_*p*_, *y*_*p*_, *z*_*p*_) = (*x*, *y*, *β*), *x* ∈ [0, ∞), *y* ∈ [*α*_1_, *α*_2_], *α*_*i*_ and *β* are real constants, and *ρ*^2^ = (*x* − *X*)^2^ + *y*^2^ + *β*^2^ (later we will set *α*_1_ = −*α* and *α*_2_ = *α*).

Let *λ* to be *λ*^2^ = (*x* − *X*)^2^ + *β*^2^ and *y* = *λ*tan*ψ*, then $\lambda^{2}+y^{2}=\lambda^{2}+\lambda^{2}\tan^{2}\psi =\lambda^{2}\sec^{2}\psi$ and $dy=\lambda \sec^{2}\psi \;d\psi$. Then the upper bound of integration *y* = *α*_2_, $\psi_{2}=\arctan[\alpha_{2}/\lambda ]$, and at the lower bound of integration at *y* = *α*_1_, $\psi_{1}=\arctan[\alpha_{1}/\lambda ]$, so that

B 1 $$E=n\int_{0}^{\infty }\lambda^{-5}\int_{\psi_{1}}^{\psi_{2}}[-A\cos^{4}\psi +\lambda^{-6}B\cos^{10}\psi ]\;d\psi \;dx.$$

The inner integral for the interaction energy can be expressed in terms of integrals of powers of cosine functions, *I*_*n*_(*λ*, *α*_*i*_). The general form of *I*_*n*_(*λ*, *α*_*i*_) is defined as

$$I_{n}(\lambda ,\alpha_{i})=\int_{0}^{\theta }\cos^{2n}\psi \;d\psi .$$

For the expression (B 1), we require *I*_2_(*λ*, *α*_*i*_) and *I*_5_(*λ*, *α*_*i*_)

$$\begin{array}{rl} I_{2}(\lambda ,\alpha_{i}) & =\int_{0}^{\theta }\cos^{4}\psi \;d\psi ,\\ & =\frac{1}{4}\frac{\alpha_{i}\lambda^{3}}{{\left(\lambda^{2}+\alpha_{i}^{2}\right)}^{2}}+\frac{3}{8}\frac{\alpha_{i}\lambda }{\left(\lambda^{2}+\alpha_{i}^{2}\right)}+\frac{3}{8}\arctan\left(\frac{\alpha_{i}}{\lambda }\right) \end{array}$$

and

$$\begin{array}{rl} I_{5}(\lambda ,\alpha_{i}) & =\int_{0}^{\theta }\cos^{10}\psi \;d\psi ,\\ & =\frac{1}{10}\frac{\alpha_{i}\lambda^{9}}{{\left(\lambda^{2}+\alpha_{i}^{2}\right)}^{5}}+\frac{9}{80}\frac{\alpha_{i}\lambda^{7}}{{\left(\lambda^{2}+\alpha_{i}^{2}\right)}^{4}}+\frac{21}{160}\frac{\alpha_{i}\lambda^{5}}{{\left(\lambda^{2}+\alpha_{i}^{2}\right)}^{3}}+\frac{21}{128}\frac{\alpha_{i}\lambda^{3}}{{\left(\lambda^{2}+\alpha_{i}^{2}\right)}^{2}}\\ & \;+\frac{63}{256}\frac{\alpha_{i}\lambda }{\left(\lambda^{2}+\alpha_{i}^{2}\right)}+\frac{63}{256}\arctan\left(\frac{\alpha_{i}}{\lambda }\right), \end{array}$$

where tan*θ* = *α*_*i*_/*λ*. Therefore, the potential energy between a point and the vertical plane at *z* = *β* is given by

$$E=n\int_{0}^{\infty }\lambda^{-5}[-A(I_{2}(\lambda ,\alpha_{2})-I_{2}(\lambda ,\alpha_{1}))+\lambda^{-6}B(I_{5}(\lambda ,\alpha_{2})-I_{5}(\lambda ,\alpha_{1}))]\;dx.$$

If *α*_2_ and *α*_1_ are equidistant from the *z*-axis, i.e. *α*_2_ = −*α*_1_ = *α*, the result can be simplified further, such that the interaction energy is given by

B 2 $$E=2n\int_{0}^{\infty }\lambda^{-5}[-AI_{2}(\lambda ,\alpha )+\lambda^{-6}BI_{5}(\lambda ,\alpha )]\;dx.$$

In what follows, we evaluate the integral of the attractive (first) term to write it in terms of standard functions. Since the process to determine an expression for the integral of the repulsive (second) term is much the same, we do not provide details here.

**B.1. Evaluation of the attractive term**

In this subsection, we determine an expression for the remaining integral of the attractive (first) component. We write

B 3*a* $$\begin{array}{rl} \int_{0}^{\infty }\lambda^{-5}I_{2}(\lambda ,\alpha )\;dx & =\int_{0}^{\infty }\lambda^{-5}\left[\frac{1}{4}\frac{\alpha \lambda^{3}}{{\left(\lambda^{2}+\alpha^{2}\right)}^{2}}+\frac{3}{8}\frac{\alpha \lambda }{\left(\lambda^{2}+\alpha^{2}\right)}+\frac{3}{8}\arctan\left(\frac{\alpha }{\lambda }\right)\right]\;dx,\\ & =\int_{\lambda_{0}}^{\infty }\left[\frac{1}{4}\frac{\alpha }{\lambda^{2}{\left(\lambda^{2}+\alpha^{2}\right)}^{2}}+\frac{3}{8}\frac{\alpha }{\lambda^{4}(\lambda^{2}+\alpha^{2})}\right]\frac{\lambda }{{\left(\lambda^{2}-\beta^{2}\right)}^{1/2}}\;d\lambda \\ & \;+\int_{0}^{\infty }\frac{3}{8}\lambda^{-5}\arctan\left(\frac{\alpha }{\lambda }\right)\;dx,\\ & =\int_{\lambda_{0}}^{\infty }\left[-\frac{1}{8\alpha^{3}}\frac{1}{\lambda }+\frac{3}{8\alpha }\frac{1}{\lambda^{3}}\right]\frac{1}{{\left(\lambda^{2}-\beta^{2}\right)}^{1/2}}\;d\lambda \end{array}$$

B 3*b* $$\;\;\;\;\;+\int_{\lambda_{0}}^{\infty }\left[\frac{1}{8\;\alpha^{3}}\frac{\lambda }{\left(\lambda^{2}+\alpha^{2}\right)}-\frac{1}{4\alpha }\frac{\lambda }{{\left(\lambda^{2}+\alpha^{2}\right)}^{2}}\right]\frac{1}{{\left(\lambda^{2}-\beta^{2}\right)}^{1/2}}\;d\lambda$$

B 3*c* $$+\int_{0}^{\infty }\frac{3}{8}\lambda^{-5}\arctan\left(\frac{\alpha }{\lambda }\right)\;dx,$$

where *λ*^2^ = (*x* − *X*)^2^ + *β*^2^ and *λ*_0_ = (*X*^2^ + *β*^2^)^1/2^. The expressions will be determined in three separate parts, as the three integrals require slightly different techniques to be evaluated. First consider equation (B 3*a*),

$$\begin{array}{rl} \int_{\lambda_{0}}^{\infty }\left[-\frac{1}{8\alpha^{3}}\frac{1}{\lambda }+\frac{3}{8\alpha }\frac{1}{\lambda^{3}}\right]\frac{1}{{\left(\lambda^{2}-\beta^{2}\right)}^{1/2}}\;d\lambda & =\int_{\theta_{0}}^{\pi /2}\left[-\frac{1}{8\alpha^{3}}\cdot \frac{1}{\beta }+\frac{3}{8\alpha }\cdot \frac{1}{\beta^{3}}\cos^{2}\theta \right]\;d\theta ,\\ & =-\frac{1}{8\alpha^{3}\beta }J_{0}(\beta ,-X)+\frac{3}{8\alpha \beta^{3}}J_{1}(\beta ,-X), \end{array}$$

where cos*θ* = *β*/*λ* and $\theta_{0}=\arctan[-X/\beta ]$. The final result can be written in terms of the definite integrals for the cosine functions, *J*_*n*_(*β*, − *X*), and the general form of is given by

$$J_{n}(\beta ,-X)=\int_{\theta }^{\pi /2}\cos^{2n}\psi \;d\psi ,$$

where tan*θ* = −*X*/*β*.

For the expression (B 3*a*), we require *J*_0_(*β*, − *X*) and *J*_1_(*β*, − *X*) which can be evaluated, respectively, as

$$J_{0}(\beta ,-X)=\frac{\pi }{2}-\left[\arctan\left(\frac{-X}{\beta }\right)\right]\;\text{and}\;J_{1}(\beta ,-X)=\frac{1}{2}\cdot \frac{\pi }{2}-\left[\frac{1}{2}\arctan\left(\frac{-X}{\beta }\right)\right].$$

Now consider expression (B 3*b*),

$$\begin{array}{rl} & \int_{\lambda_{0}}^{\infty }\left[\frac{1}{8\alpha^{3}}\frac{\lambda }{\left(\lambda^{2}+\alpha^{2}\right)}-\frac{1}{4\alpha }\frac{\lambda }{{\left(\lambda^{2}+\alpha^{2}\right)}^{2}}\right]\frac{1}{{\left(\lambda^{2}-\beta^{2}\right)}^{1/2}}\;d\lambda \\ & \;=\int_{w_{0}}^{\infty }\left[\frac{1}{8\alpha^{3}}\cdot \frac{1}{w}-\frac{1}{4\alpha }\cdot \frac{1}{w^{3}}\right]\frac{1}{{\left(w^{2}-(\alpha^{2}+\beta^{2})\right)}^{1/2}}\;dw, \end{array}$$

where *w*^2^ = *λ*^2^ + *α*^2^ and *w*_0_ = (*α*^2^ + *β*^2^ + *X*^2^)^1/2^. The resulting integral in terms of *w* can be further transformed into an integrand of cosine functions, such that

$$\begin{array}{rl} & \int_{w_{0}}^{\infty }\left[\frac{1}{8\alpha^{3}}\cdot \frac{1}{w}-\frac{1}{4\alpha }\cdot \frac{1}{w^{3}}\right]\frac{1}{{\left(w^{2}-(\alpha^{2}+\beta^{2})\right)}^{1/2}}\;dw\\ & \;=\int_{\theta_{0}}^{\pi /2}\left[\frac{1}{8\alpha^{3}}\cdot \frac{1}{{\left(\alpha^{2}+\beta^{2}\right)}^{1/2}}-\frac{1}{4\alpha }\cdot \frac{1}{{\left(\alpha^{2}+\beta^{2}\right)}^{3/2}}\cos^{2}\theta \right]\;d\theta ,\\ & \;=\frac{1}{8\alpha^{3}{\left(\alpha^{2}+\beta^{2}\right)}^{1/2}}J_{0}(\sqrt{\alpha^{2}+\beta^{2}},-X)-\frac{1}{4\alpha {\left(\alpha^{2}+\beta^{2}\right)}^{3/2}}J_{1}(\sqrt{\alpha^{2}+\beta^{2}},-X), \end{array}$$

where $\cos\theta =\sqrt{\alpha^{2}+\beta^{2}}/w$ and $\theta_{0}=\arctan[-X/\sqrt{\alpha^{2}+\beta^{2}}]$. The final result can be written in terms of the definite integrals for the cosine functions, $J_{n}(\sqrt{\alpha^{2}+\beta^{2}},-X)$.

Finally consider expression (B 3*c*),

$$\begin{array}{rl} & \int_{0}^{\infty }\frac{3}{8}{\left({\left(x-X\right)}^{2}+\beta^{2}\right)}^{-5/2}\arctan\left(\frac{\alpha }{{\left({\left(x-X\right)}^{2}+\beta^{2}\right)}^{1/2}}\right)\;dx=\frac{3}{8\alpha^{3}}\int_{0}^{w_{0}}\frac{w^{3}\arctan w}{{\left(\alpha^{2}-\beta^{2}w^{2}\right)}^{1/2}}\;dw, \end{array}$$

where $w=\alpha /\sqrt{{\left(x-X\right)}^{2}+\beta^{2}}$ and $w_{0}=\alpha /\sqrt{\beta^{2}+X^{2}}$.

$$\begin{array}{rl} & \frac{3}{8\alpha^{3}}\int_{0}^{w_{0}}\frac{w^{3}\arctan w}{{\left(\alpha^{2}-\beta^{2}w^{2}\right)}^{1/2}}\;dw\\ & \;=-\frac{3}{8\alpha^{3}\beta^{2}}\int_{0}^{w_{0}}w{\left(\alpha^{2}-\beta^{2}w^{2}\right)}^{1/2}\arctan w\;dw\\ & \;+\frac{3}{8\alpha \beta^{2}}\int_{0}^{w_{0}}w{\left(\alpha^{2}-\beta^{2}w^{2}\right)}^{-1/2}\arctan w\;dw,\\ & \;=-\frac{3}{8\alpha^{3}\beta^{2}}\left[{-\frac{{\left(\alpha^{2}-\beta^{2}w^{2}\right)}^{3/2}}{3\beta^{2}}\arctan w|}_{0}^{w_{0}}+\frac{1}{3\beta^{2}}\int_{0}^{w_{0}}\frac{{\left(\alpha^{2}-\beta^{2}w^{2}\right)}^{3/2}}{1+w^{2}}\;dw\right]\\ & \;+\frac{3}{8\alpha \beta^{2}}\left[{-\frac{{\left(\alpha^{2}-\beta^{2}w^{2}\right)}^{1/2}}{\beta^{2}}\arctan w|}_{0}^{w_{0}}+\frac{1}{\beta^{2}}\int_{0}^{w_{0}}\frac{{\left(\alpha^{2}-\beta^{2}w^{2}\right)}^{1/2}}{1+w^{2}}\;dw\right],\\ & \;=-\frac{{\left(\alpha^{2}-\beta^{2}w_{0}^{2}\right)}^{1/2}}{8\alpha^{3}\beta^{4}}(2\alpha^{2}+\beta^{2}w_{0}^{2})\arctan w_{0}\\ & \;+\frac{1}{8\alpha^{3}\beta^{4}}\int_{0}^{w_{0}}\frac{{\left(\alpha^{2}-\beta^{2}w^{2}\right)}^{1/2}(2\alpha^{2}+\beta^{2}w^{2})}{1+w^{2}}\;dw,\\ & \;=-\frac{{\left(\alpha^{2}-\beta^{2}w_{0}^{2}\right)}^{1/2}}{8\alpha^{3}\beta^{4}}(2\alpha^{2}+\beta^{2}w_{0}^{2})\arctan w_{0}+\frac{1}{8\alpha^{3}\beta^{2}}\int_{0}^{w_{0}}{\left(\alpha^{2}-\beta^{2}w^{2}\right)}^{1/2}\;dw\\ & \;+\frac{\left(2\alpha^{2}-\beta^{2}\right)}{8\alpha^{3}\beta^{4}}\int_{0}^{w_{0}}\frac{{\left(\alpha^{2}-\beta^{2}w^{2}\right)}^{1/2}}{1+w^{2}}\;dw. \end{array}$$

To evaluate the remaining integrals, let *βw* = *α*sin*θ* and $\theta_{0}=\text{arccos}[-X/{\left(\beta^{2}+X^{2}\right)}^{1/2}]$, then

$$\begin{array}{rl} & \int_{0}^{\infty }\frac{3}{8}{\left({\left(x-X\right)}^{2}+\beta^{2}\right)}^{-5/2}\arctan\left(\frac{\alpha }{{\left({\left(x-X\right)}^{2}+\beta^{2}\right)}^{1/2}}\right)\;dx\\ & \;=-\frac{{\left(\alpha^{2}-\beta^{2}w_{0}^{2}\right)}^{1/2}}{8\alpha^{3}\beta^{4}}(2\alpha^{2}+\beta^{2}w_{0}^{2})\arctan w_{0}+\frac{1}{8\alpha \beta^{3}}\int_{0}^{\theta_{0}}\cos^{2}\theta \;d\theta \\ & \;+\frac{\left(2\alpha^{2}-\beta^{2}\right)}{8\alpha \beta^{5}}\int_{0}^{\theta_{0}}\frac{1}{1+k^{2}\tan^{2}\theta }\;d\theta ,\\ & \;=\frac{1}{8\beta^{4}}\frac{X(3\beta^{2}+2X^{2})}{{\left(\beta^{2}+X^{2}\right)}^{3/2}}\arctan\left(\frac{\alpha }{{\left(\beta^{2}+X^{2}\right)}^{1/2}}\right)+\frac{1}{8\alpha \beta^{3}}G_{0}(-X,\beta )\\ & \;+\frac{2\alpha^{2}-\beta^{2}}{8\alpha^{3}\beta^{4}}\left[{\left(\beta^{2}+\alpha^{2}\right)}^{1/2}\arctan\left(\frac{{\left(\beta^{2}+\alpha^{2}\right)}^{1/2}}{-X}\right)-\beta \arctan\left(\frac{\beta }{-X}\right)\right], \end{array}$$

where *k*^2^ = 1 + (*α*/*β*)^2^ and *G*_0_( − *X*, *β*) is defined as a trigonometric integral

$$G_{m}(-X,\beta )=\int_{0}^{\theta }\sin^{2m}\psi \cos^{2}\psi \;d\psi ,$$

where tan*θ* = *β*/−*X*, and where *G*_0_(− *X*, *β*) is given by

$$\begin{array}{rl} G_{0}(-X,\beta ) & =\int_{0}^{\theta }\cos^{2}\psi \;d\psi \\ & =-\frac{1}{2}\frac{\beta X}{\left(X^{2}+\beta^{2}\right)}+\frac{1}{2}\text{arccos}\left(\frac{-X}{{\left(X^{2}+\beta^{2}\right)}^{1/2}}\right). \end{array}$$

## References

[1] Stevens K, Thamwattana N, Tran-Duc T. 2022 Continuum modeling with functional Lennard-Jones parameters for methane storage inside various carbon nanostructures. ACS Omega **7**, 29 773-29 786. (10.1021/acsomega.2c02485) PMC9434623 36061669

[2] Stephan S, Langenbach K, Hasse H. 2019 Interfacial properties of binary Lennard-Jones mixtures by molecular simulation and density gradient theory. J. Chem. Phys. **150**, 174704. (10.1063/1.5093603) 31067907

[3] Alshehri MH. 2021 Continuum modelling for encapsulation of anticancer drugs inside nanotubes. Mathematics **9**, 2469. (10.3390/math9192469)

[4] Adisa OO, Cox BJ, Hill JM. 2011 Encapsulation of methane molecules into carbon nanotubes. Physica B **406**, 88-93. (10.1016/j.physb.2010.10.027)

[5] Baowan D, Thamwattana N. 2011 Modelling adsorption of a water molecule into various pore structures of silica gel. J. Math. Chem. **49**, 2291-2307. (10.1007/s10910-011-9887-3)

[6] Baowan D, Hill JM. 2016 Mathematical modeling of interaction energies between nanoscale objects: a review of nanotechnology applications. Adv. Mech. Eng. **8**, 1-16. (10.1177/1687814016677022)

[7] Louw KI, Bradshaw-Hajek BH, Hill JM. 2022 Interaction of ferric ions with europium metal organic framework and application to mineral processing sensing. Philos. Mag. **102**, 1231-1246. (10.1080/14786435.2022.2061066)

[8] Thamwattana N, Sarapat P, Chan Y. 2019 Mechanics and dynamics of lysozyme immobilisation inside nanotubes. J. Phys.: Condens. Matter **31**, 265901. (10.1088/1361-648X/ab13c9) 30917355

[9] Lennard-Jones JE. 1931 Cohesion. Proc. Phys. Soc. **43**, 461-482. (10.1088/0959-5309/43/5/301)

[10] Lennard-Jones JE, Devonshire AF. 1938 Critical phenomena in gases. II. Vapour pressures and boiling points. Proc. R. Soc. Lond. A **165**, 1-11. (10.1098/rspa.1938.0039)

[11] Cox BJ, Thamwattana N, Hill JM. 2007 Mechanics of atoms and fullerenes in single-walled carbon nanotubes. I. Acceptance and suction energies. Proc. R. Soc. A **463**, 461-477. (10.1098/rspa.2006.1771)

[12] Xu H, Dong Y, Wu Y, Ren W, Zhao T, Wang S, Gao J. 2018 An-OH group functionalized MOF for ratiometric Fe^3+^ sensing. J. Solid State Chem. **258**, 441-446. (10.1016/j.jssc.2017.11.013)

[13] Rozenberga L, Skinner W, Lancaster DG, Bloch WM, Blencowe A, Krasowska M, Beattie DA. 2022 A europium metal-organic framework for dual Fe^3+^ ion and pH sensing. Sci. Rep. **12**, 11982. (10.1038/s41598-022-15663-z) 35835797 PMC9283444

[14] Jarzecki AA, Anbar AD, Spiro TG. 2004 DFT analysis of $\mathrm{Fe}{\left(H_{2}O\right)}_{6}^{3+}$ and $\mathrm{Fe}{\left(H_{2}O\right)}_{6}^{2+}$ structure and vibrations; Implications for isotope fractionation. J. Phys. Chem. A **108**, 2726-2732. (10.1021/jp036418b)

[15] Rappé AK, Casewit CJ, Colwell KS, Goddard III WA, Skiff WM. 1992 UFF, a full periodic table force field for molecular mechanics and molecular dynamics simulations. J. Am. Chem. Soc. **114**, 10 024-10 035. (10.1021/ja00051a040)

